# How COVID-19 exposed pre-existing roadblocks for cancer control in Africa: strategies, lessons and recommendations from the 2019–2020 Africa Cancer Research and Control ECHO

**DOI:** 10.3332/ecancer.2022.1516

**Published:** 2023-03-06

**Authors:** Annet Nakaganda, Nwamaka Lasebikan, Elise M Garton, Benda Kithaka, Eunice Garanganga, Alicia A Livinski, Mishka K Cira

**Affiliations:** 1 Uganda Cancer Institute, PO Box 3935, Kampala, Uganda; 2 University of Nigeria Teaching Hospital, 8F26+HQ2, Enugu 402109, Nigeria; 3 Center for Global Health, National Cancer Institute, National Institutes of Health, 9609 Medical Center Drive, Rockville, MD 20850, USA; 4 KILELE Health Association, PO Box 1627, Nairobi, Kenya; 5 Hospice and Palliative Care Association of Zimbabwe, 13 Lezard Avenue, Milton Park, Harare, Zimbabwe; 6 National Institutes of Health Library, Office of Research Services, NIH, 10 Center Drive Building 10, Room 1L-25, MSC 1150, Bethesda, MD 20892, USA

**Keywords:** COVID-19, pandemic, cancer, control, Africa, strategies, lessons, recommendations

## Abstract

**Background:**

The COVID-19 related mitigation measures adversely affected various cancer control activities in Africa, with cancer prevention and screening activities amongst the most significantly impacted. When the COVID-19 pandemic struck, the Africa Cancer Research and Control ECHO utilised their virtual platform to share experiences and knowledge of how to continue cancer service delivery during the pandemic. This analysis describes the evolved strategies, dilemmas, and recommendations to strengthen the health systems for cancer control in Africa.

**Methods:**

Eleven 1-hour-long sessions about the then newly emerging coronavirus infection and its impact on cancer control in Africa were held from April 2020 to August 2020, using Zoom®. An average of 39 participants attended the sessions including scientists, clinicians, policymakers and global partners. Sessions were analysed thematically.

**Results:**

Most strategies to maintain cancer services during the COVID-19 pandemic centred around cancer treatment, with few strategies on maintaining cancer prevention services, early detection, palliative care and research services. The most mentioned challenge during the pandemic was fear of exposure to COVID-19 infection at the health facility during diagnosis, treatment or follow-up for cancer care. Other challenges were disruptions to service delivery, inaccessibility of cancer treatment, disruption of research activities and a lack of psychosocial support for COVID-19 related fear/anxiety. Significantly, this analysis shows that the COVID-19 related mitigation measures exacerbated existing predicaments in Africa, such as inadequate attention to cancer prevention strategies, psychosocial and palliative services and cancer research. The Africa Cancer ECHO recommends African countries to leverage the infrastructure developed in response to COVID-19 pandemic to strengthen the health system along the entire cancer control continuum. This calls for urgent action to develop and implement evidence-based frameworks and comprehensive National Cancer Control Plans that will withstand any future disruptions.

## Background

In 2020, the SARS-CoV-2 viral pandemic (COVID-19 pandemic) caused many African countries to implement restrictions to prevent the spread of the virus and development of disease (COVID-19) [1]. In reviewing the literature, most of the COVID-19 pandemic responses in Africa were based on the World Health Organization (WHO) issued guidance, but varied depending on the number of cases in particular countries and attitudes and beliefs of the leaders about COVID-19 infection and health [2, 3]. To adhere to the WHO COVID-19 prevention measures and manage the shift of health worker focus to COVID-19 associated tasks, many health facilities and cancer centres re-organised or suspended some cancer services [2, 4]. Currently, there is little data available on the impact of the COVID-19 pandemic on African cancer patients. But as of November 2022, 12 million cases of COVID-19 were recorded and overall deaths due to COVID-19 reached 257,984 in Africa [5–8].

Here we describe real-life experiences and accounts of how the COVID-19 pandemic impacted cancer control services in the African region, the different ways these impacts were addressed, lessons learned and recommendations on building further resilience into healthcare systems as shared by participants in the Africa Cancer ECHO COVID-19 series.

## Methods

When the COVID-19 pandemic struck, the Africa Cancer ECHO utilised their virtual platform to share experiences and knowledge of continuing cancer service delivery during the pandemic. The Africa Cancer ECHO is an African cancer expert-led initiative that regularly convenes cancer control experts to discuss the implementation of evidence-based cancer control initiatives in Africa [9].

From April 2020 to August 2020, the Africa Cancer ECHO held 11 1-hour-long weekly (In April 2020) and bi-weekly (May 2020 to August 2020) sessions about the then newly emerging SARS-CoV-2 pandemic, preventive measures and its impact on cancer control services. Sessions were held on the web-based virtual meeting platform, Zoom^®^ and were recorded, with participant knowledge and consent. Each session was attended by an average of 39 participants including regional scientists, clinicians, advocates, planners, policymakers and global partners.

The sessions used Project ECHO’s bi-directional learning model to present country-level cases on locally generated initiatives and dilemmas experienced in cancer control during the COVID-19 pandemic (Table 1) [10]. The country cases were followed by didactic presentations on relevant effective practices to the topic, and discussions amongst the participants of locally relevant recommendations for cancer control during the pandemic. The audio recordings of the sessions were transcribed verbatim by an experienced non-attendee. Data were analysed thematically using a hybrid deductive-inductive approach [11].

First, three authors (MKC, EG, AN) used deductive coding to define five Level I codes and ten Level II codes based on their knowledge of the session content (Table 2). Next, MKC and EG independently coded the session transcripts based on the pre-selected Level I codes using QDA Miner Lite and Microsoft Excel software. The text was further defined deductively by four authors (MKC, EG, AN, NL) who individually coded the text along the cancer control continuum [12]. This formed the Level III codes (Table 3).

Two authors (MKC, EG) used inductive coding and independently coded text from Levels I, II and III to specify the content of the sessions and this coding added a Level IV code. After completing all the coding, the resulting themes and codes were reviewed, discussed and agreed upon by all the authors.

### Ethics consideration

Ethical clearance for publishing the results of our work was received from Uganda Cancer Institute, University of Nigeria Teaching Hospital, and National Institutes of Health, USA. In addition, verbal informed consent to record the ECHO sessions was always sought from the participants before each session. The audio recordings of the sessions were anonymously transcribed verbatim by an experienced non-attendee. Confidentiality was maintained by de-identifying the transcripts and use of codes on the transcripts.

## Results

### Characteristics of the ECHO participants

A total of 160 participants registered for the Africa Cancer ECHO between 2019 and 2020 and attended the COVID-19 ECHO series at an average attendance of about 39 participants per session. These participants were from a range of countries and organisations/institutions across the world. Appendix provides details of the geographical/country distribution of the participants spanning 23 countries/institutions globally. Also, participants had varied experience and expertise in cancer control (Table 2) including: cancer advocacy (15%), government/policy maker (25%), health worker (11%), researcher (29%), United Nations Agency (5%) and technical advisors (15%).

### Main themes from the COVID-19 cancer control experience

The analysis identified five overarching thematic areas including: strategies for cancer control during COVID-19 pandemic in Africa; impact of COVID-19 pandemic on cancer control services; challenges related to COVID-19 pandemic mitigation measures; unresolved dilemmas for cancer control in Africa; and recommendations for cancer control during and after the COVID-19 pandemic (Tables 3–5). We consolidated the three themes (COVID-19 impact, related challenges and unresolved dilemmas) together for easier presentation of the results and discussion sections. Table 6 provides a summary of the findings by major thematic areas.

### Strategies for cancer control during COVID-19 pandemic in Africa

**Figure 1.**
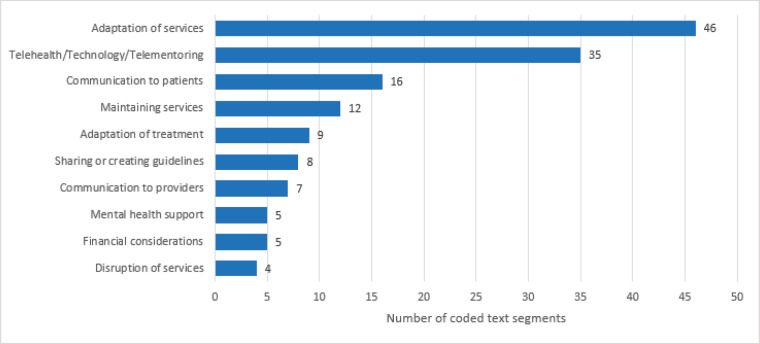
Ten most common strategies for maintaining cancer services in Africa, during the COVID-19 pandemic.

Across countries and health facilities, strategies emphasised adaptation of service delivery models to maintain cancer services; use of telehealth for patient communication; and development, modification and sharing of cancer treatment protocols (Figure 1). Adaptation of cancer services meant modification of service delivery models to suit the COVID-19 prevention measures and restrictions, for example reconfiguring chemotherapy infusion centres and patient waiting areas to allow social distancing, increasing psychosocial support to allay patients’ fear and anxiety and provision of cancer treatment at local and lower-level facilities to minimise patient travel to cancer centres. Also, cancer centres developed and/or modified treatment protocols or workflows that were quickly adapted as more information about COVID-19 infection became available. Other strategies involved a transition from in-person care to telehealth (including online multidisciplinary tumour boards) and the need for consistent messaging. This facilitated increased communication with patients and addressed the challenges of a lack of information or misinformation about COVID-19 pandemic. In the whole process, health workers developed an understanding of the requirements of an effective communication strategy for telehealth as one participant narrated that effective telecommunication is to:

“*Provide context, consideration, caring, and commitment. When we say to the patient, that we might be changing something or delaying something, [for example] we can’t screen right now; the ‘context’ is ‘sharing the why’ we’re doing this. The ‘consideration’ is to highlight how you came to the decisions. The ‘caring’ is to express your concern and recognize the patient’s emotions and acknowledge that. And then the ‘commitment’ that; the difficulty here is the uncertainty of how long this is going to last, but through all this I’m going to be your doctor*” (11 June 2020 ECHO participant).

Many strategies were shared with the understanding that the COVID-19 pandemic and its impact on society and healthcare services are likely to be long-lasting. For example, a presenter shared the importance of maintaining chemotherapy for cancer patients:

*‘We cannot afford to interrupt any treatment at this stage. If we do so, actually the implication is much more disastrous than the pandemic itself. These are things that we have made sure all health workers understand and take into consideration, not only the health workers or hospital managers, but also the leadership in the hospital and the hospital administrations’ *(6 August 2020 ECHO participant).

**Figure 2.**
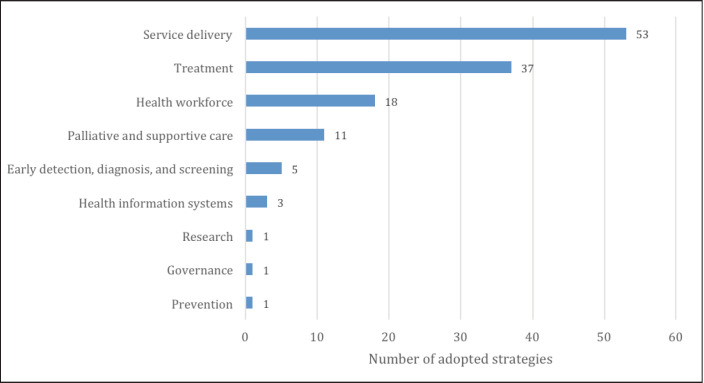
Number of strategies (by element of cancer control continuum) for maintaining cancer services in Africa during COVID-19 pandemic.

The analysis also grouped the strategies according to cancer control continuum and strategies identified were primarily related to service delivery (53), treatment (37) and health workforce development (18), with few strategies mentioned on cancer prevention, early detection and research (Figure 2).

### COVID-19 impact, challenges and unresolved dilemmas for cancer control

**Figure 3.**
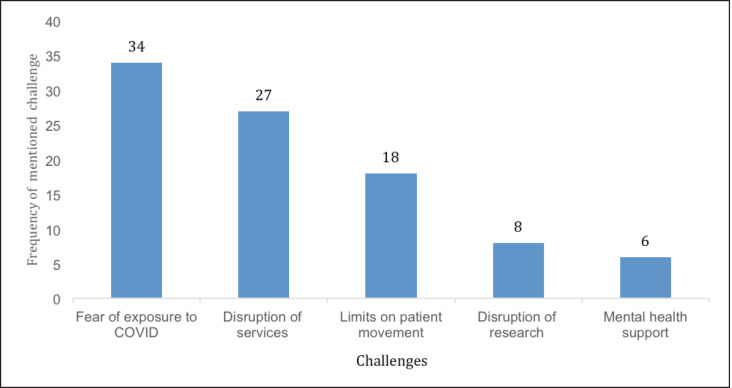
Five most common challenges faced during COVID-19 pandemic.

The most commonly reported challenge was the fear of exposure to COVID-19 infection (by both patients and health workers) at the health facility during diagnosis, treatment or follow-up for cancer care (Figure 3). Health workers’ fear of exposure to COVID-19 infection was mainly due to a lack of personal protective equipment (PPE); crowded and congested hospital space and a lack of readily accessible washing facilities. One participant narrated that:

*‘A third area that is a challenge is limited PPE and that is something that has been discussed many times already on the platform. We have routinely had gloves available, but masks and certainly eye shields is not something that we routinely use’* (9th April 2020)

And another participant added that:

*‘Our physical infrastructure has also posed some challenges with implementing good infection control, especially with the lack of sinks on the wards/clinics; congestion and areas from where we administer chemotherapy are quite crowded. We have open wards here and do not have a lot of room to do social distancing and isolate patients with respiratory symptoms’ *(9th April 2020).

Related challenges focused on COVID-19 related anxiety and stigma; a lack of psychosocial support services and ultimately patient inaccessibility to cancer services. One participant indicated that:

‘*One of the problems that we see at the hospitals is that the hospitals are empty. Patients are scared of going to the hospitals. Most would rather stay at home with a huge lump on their breast than to go to the hospital, fearing they would contract coronavirus (COVID-19 infection) at the hospital*’ (23 July 2020 ECHO participant).

Another commonly reported challenge was the overall disruption of cancer services spanning the full cancer control continuum from prevention to early detection, diagnosis and treatment, to cancer surveillance and research. Reasons reported for disruptions in cancer services were COVID-related travel restrictions, compromised supply chain, limited access to health facilities, a lack of electronic data collection systems and diversion of already scarce resources to the COVID-19 response. For example, cancer screening services and most surgeries were put on hold. One participant narrated that:

*‘We have cancelled all cancer screening visits, we converted the space that we had previously used for routine cancer screening to become our COVID-19 screening centre; we are deferring all of our follow ups as needed, and actually most surgeries have been cancelled from the anaesthesiologist standpoint of view’ *(9th April 2020).

Although some services could be offered virtually, use of telehealth still came with challenges around patient discomfort with telehealth and technological limitations such as slow bandwidth, connectivity and cost of mobile airtime. In addition, health workers expressed inadequate skills and capacity in use of telehealth and telecommunication, for example, a lack of skills for assessing psychosocial needs of patients through telehealth was raised as one participant accounted that;

*“One of the palliative care nurses said to me that ‘when assessing pain and symptoms, I use my eyes, my ears, touch, smell, etc., but now I’m not confident, relying on only a phone call’” *(21 May 2020 ECHO participant).

There were also significant concerns about the pandemic’s impact on cancer research activities and research funding, particularly in public institutions, and not-for-profit organisations that struggled to maintain financial solvency due to the economic impact of the pandemic. Cancer research activities in Africa were widely stopped during the COVID-19 pandemic. For example, cancer registration in many facilities stopped as it relied on manual, in-person and paper-based case finding, which was not feasible due to safety concerns and lockdown measures. One of the cancer registrar discussed that:

*‘Of course, it’s an open secret that the whole world is panicking about this pandemic. But we as cancer registrars have never thought of how our surveillance officers are affected. Clerk stations in the hospitals are very small and overcrowded. So the clerks are now working in shifts, but our surveillance officers are not considered for shifts because we are not part of the hospital staff’* (23 July 2020 ECHO participant)

However, some participants recognised the positive impact of the COVID-19 pandemic like enhancement of digital platforms in cancer care, telehealth, drone drug delivery and reliance on radiotherapy as opposed to surgical interventions. Similarly, simple strategies for infection control such as sanitising working stations in hospital wards had a positive impact on overall hospital hygiene. Overall, the widespread approach to COVID-19 prevention demonstrated the importance of multisector approach to disease control as one presenter said that:

*‘If we look at what COVID has taught us, it is possible for the global community to rally around a cause to bring the necessary resources, necessary political willingness, and necessary policies to make changes in the healthcare system if an issue has gotten the high-level uptake on advocacy’* (5/28/2020 ECHO participant).

### Recommendations for cancer control during and after the COVID-19 pandemic

**Figure 4.**
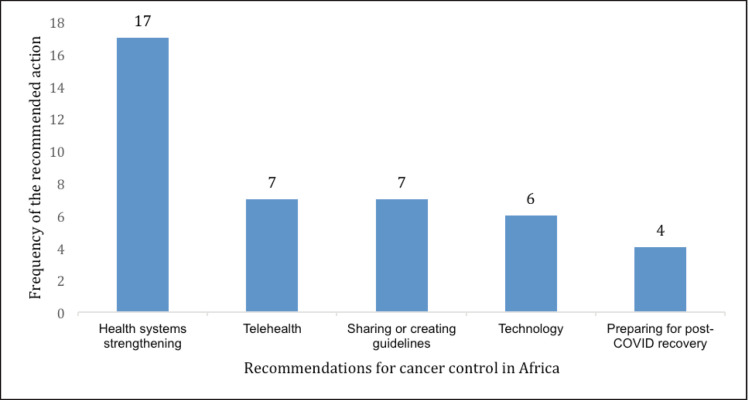
Recommendations for cancer control during and after COVID-19 pandemic in Africa.

Recommendations for responding to COVID-19-related challenges focused primarily on health system strengthening, particularly integrating and decentralising cancer control services in all health care service levels and health service structures (Figure 4). Additionally, participants recommended the continued and increased use of digital technology for cancer control and creating and sharing guidelines for adapting clinical practice in Africa. Participants emphasised the importance of using the momentum and government support in addressing the COVID-19 pandemic to increase investment in cancer control, and leveraging the infrastructure developed in response to the pandemic to further strengthen cancer response and health systems in their countries.

*‘...whilst infrastructure for COVID is being built, you can use that same infrastructure for cancer services. Things like decentralization of services, because I’ve noticed that the HIV community in my country, a lot of the patients are decentralized up to the village level, and if*

## Discussion

Based on a thematic analysis of the presentations and discussions held during the early months of the COVID-19 pandemic by the Africa Cancer ECHO, this study examined the strategies and impact of COVID-19 on cancer control activities in Africa and highlighted the dilemmas, lessons and recommendations for sustaining cancer control strategies during the current and future pandemics.

The results of this analysis show that many of the presented strategies and initiatives for sustaining cancer services during the pandemic were centred around cancer treatment services. Consistent with the literature, there were very few strategies that focused on other elements of cancer control such as cervical cancer screening (a leading cause of cancer deaths in Africa), prevention, early detection and palliative care services [9, 13]. These findings match with those observed in earlier studies that cancer screening, prevention, palliative care and psychosocial support services were completely suspended in many African countries during the pandemic [8–10, 14]. This observation may be an important indicator of the misalignment between where resources for cancer control actually go and where resources should go to have the greatest long-term impact on cancer control in Africa. Literature shows that most cancer deaths in Africa are attributed to delays in cancer screening, a lack of cancer awareness and poor healthcare systems. This is compounded by other competing healthcare priorities such as communicable diseases that limit attention to cancer prevention [15]. This implies that the strategies to control the COVID-19 pandemic exposed and exacerbated pre-existing roadblocks for cancer control in Africa.

Limited attention to cancer prevention, screening and early detection services in Africa is a big challenge and contributes greatly to late-stage cancers and cancer deaths in this region [16]. There is an urgent need to use the experience of the pandemic as a call for developing and strengthening health systems in Africa, that embrace all elements of cancer control, from prevention to palliation. This new comprehensive approach must be backed by rigorous interdisciplinary research; a strong leadership structure; partnership formation and stakeholder involvement; advocacy; and cancer control policies that set out priorities for cancer control interventions regularly.

Another recurrent theme in the Africa Cancer ECHO discussions that significantly affected cancer control activities during the pandemic was ‘fear of exposure’ to COVID-19 infection by both patients and health care providers. These findings are consistent with studies conducted in Ghana, Morocco and South Africa that noted a significant decline in patient admissions due to fear of contracting COVID-19 in hospitals [4, 16–19]. Although fear of exposure to infection became very pronounced during the COVID-19 pandemic, health care-associated infections (HCAIs) are an ever-growing public health problem and remain a dilemma in developing countries [14, 20, 21]. While exact data to estimate the burden of healthcare acquired COVID-19 infection in Africa are lacking, hospital acquired infection rates are at least two to three times higher in low- and middle-income countries than in high-income countries [20, 22, 23]. HCAIs are preventable and some of the most effective preventive measures are low-technology measures including: regular education and training of health workers; re-enforcing infection control policies and ensuring compliance; using infection-control officers; incorporating mandatory infection screening and vaccination programmes in oncology centres as a standard of care; and mandatory surveillance and reporting of HCAIs by hospitals [14, 20, 21]. The Africa Cancer ECHO recommends implementation of such long-term public health infection control measures that embrace practical evidence-based approaches. Hence, leveraging strategies created during COVID-19 pandemic will ensure sustainability of safer hospital environments, maintenance of health service delivery and possibly prevent future disruptions of cancer services due to disease pandemic.

A further tight spot expressed during the sessions was the impact of COVID-19 restrictions on cancer research activities. Participants noted the decreased availability of overall research funding for cancer control and COVID-19 research in Africa; both of which negatively impacted the researchers’ ability to collect basic epidemiological data on the impact of COVID-19 on cancer patients. This finding coincides with other observations that cancer research in Africa was substantially scaled down during the COVID-19 pandemic, and that there were smaller investments in COVID-19 funded research in Africa compared to high-income countries [16, 24]. Limited availability of research evidence and cancer data in Africa negatively impacts the implementation of well-conceived cancer control strategies. As a result, cancer control interventions in Africa are rarely based on local data or assessed to determine if they work as well in African settings. A lack of local cancer data is one of the pre-existing roadblocks exacerbated by the pandemic. The Africa Cancer ECHO urges African governments to invest in the development of routine patient-level databases to guide planning and cancer care service delivery [25].

Additionally, African governments need to embrace and committee funds for operational research to produce locally generated and timely evidence to guide health care and policy decisions, especially during pandemics [25].

### Strengths and limitations

The Africa Cancer ECHO platform is well-regarded for using regional and global expertise in cancer control and embracing diversity amongst its participants in terms of disciplines, background and health care settings. The sessions on COVID-19 and cancer control were extremely timely and needed as they ensured the rapid sharing, interpretation and understanding of new knowledge, experiences and practices, on responding to the evolving COVID-19 pandemic. However, the findings and recommendations summarised here are based on the qualitative analysis of real-world experience, knowledge shared and participants’ discussions held during the early months of COVID-19 pandemic. In addition, the composition of the Africa Cancer ECHO is dominated by the countries from sub-Saharan region (Appendix) and these findings may be different from other Africa regions (such as Northern African countries) with more resilient health systems. A more rigorous pragmatic and quantitative validation to help inform the best approaches to prevent disruption of cancer services in future pandemics is likely still needed. Hence, these findings are extremely useful in providing a starting point for future activities and investigation.

## Conclusion

The COVID-19 pandemic mitigation measures greatly disrupted cancer control services in the African region. However, the COVID-19 pandemic exposed some pre-existing predicaments in cancer control including: poor infection control practices, a paucity of data (including cancer data), inadequate levels of cancer research funding and limited attention to cancer prevention, early detection, screening and palliative care services. The Africa Cancer ECHO recommends a renewed emphasis on strengthening health systems through the development of National Cancer Control Plans (NCCPs) that are evidence-based, well-funded and resourced, and strongly managed to form a robust and sturdy health infrastructure that will withstand future disruptions from any source.

## Conflict of interest

The authors declare that they have no conflict of interest.

## Funding

This study did not receive any formal funding grant. The US National Cancer Institute funded the transcription of sessions. No other specific funding was received for the conduct of this study.

## Figures and Tables

**Table 1.** Session topics discussed in the Africa cancer ECHO COVID-19 series.

Session date	Session topic
9 April 2020	Best practices in cancer care in the COVID-19 era
30 April 2020	Cancer research and COVID-19: impact on cancer prevention and participation in research
7 May 2020	Cancer care planning: managing patient care during the pandemic
14 May 2020	Adapting cancer centre clinical protocols during the pandemic
21 May 2020	Palliative and end-of-life considerations for patients during the pandemic
28 May 2020	Challenges for cancer research dissemination and programme scale-up in the COVID-19 era
11 June 2020	Support for cancer patients and healthcare workers during the pandemic
9 July 2020	Cancer care in countries with no treatment capacity
23 July 2020	Impact of the pandemic on cancer surveillance and cancer registry implementation
6 August 2020	Country strategies for reopening cancer care services amidst the on-going pandemic
20 August 2020	Impact of COVID-19 on cancer research (short- and long-term implications)

**Table 2.** Session topics discussed in the Africa cancer ECHO COVID-19 series.

Type of occupation	Number of participants
Cancer advocate/NGO	24
Government/Ministry of Health/policy maker	40
Healthcare worker	18
Researcher	47
UN Agency	8
Technical advisor to Government/Ministry of Health	23
Grand total	160

**Table 3.** Code level I and code level II codes used in deductive coding.

Code level I	Code level II
Strategies for cancer control during COVID-19 pandemic	Strategies for controlling COVID-19 infection Strategies for maintaining cancer services during COVID-19 pandemic
Impact (implications of strategies)	Positive implications Negative implications
COVID-19 related challenges	Fear/stigma of providers Fear/stigma of patients Patient access to treatment Service delivery disruption
Unresolved dilemmas	Unresolved dilemmas for cancer control
Recommendations	Recommendations for strengthening cancer control services

**Table 4.** Code level III codes used in deductive coding.

Level III codes	Definition used
Prevention	Behavioural/environmental risk factors, health promotion, public awareness, role of primary health care, vaccination
Early detection, diagnosis and screening	Screening programmes, referral networks, pathology services, diagnostic guidelines
Treatment	Surgery, chemotherapy, radiation services, essential medicines and devices, multi-disciplinary approach (tumour boards)
Palliative and supportive care	Psychosocial/non-physical support, family/caregiver support, rehabilitation, survivorship, long-term care, end-of-life care
Service delivery	Staff/patient safety, patient navigation, healthcare delivery facilities, quality control, supply chain, infrastructure
Governance	Accountability, regulations, leadership, partnerships, monitoring and evaluation
Health workforce	Human resources, workforce development and training
Health information systems	Electronic medical records, telemedicine
Research	Cancer registry/surveillance, research funding and clinical trials
Finance	Costing, financing plan, health insurance, financial protection

**Table 5.** Code level IV codes used in inductive coding.

Level IV codes	
Adaptation of services Modification of treatment Communication to patients Communication to policymakers Communication to providers COVID-19 risk to cancer patients Disruption of research Disruption of services Exacerbation of existing disparity Fear of exposure to COVID-19 Financial considerations Health systems strengthening Isolation	Lack of protective measures Limits on patient movement Long-term impacts Maintaining services Mental health support Partnerships Patient engagement Preparing for post-COVID recovery Sharing or creating guidelines Technology Telehealth Tele-mentoring

**Table 6.** Summary of the major findings; strategies and impact, challenges and recommendations for cancer control in Africa.

Theme	Sub-theme	Activities	Impact/outcome
Strategies for cancer control during COVID-19 pandemic	Adaptation of service delivery models to maintain cancer services	Use of telehealth, technology and tele-mentoring	Health workers developed a joint understanding and appreciation of effective telehealth communication in cancer care Enhancement of digital platforms in cancer care, telehealth and drone drug delivery
		Developing, modifying and sharing cancer treatment protocols	Improved knowledge sharing and standardisation of treatment protocols Reliance on radiotherapy as opposed to surgical interventions
		Reconfiguring chemotherapy infusion centres, screening space and patient waiting areas.	Screening services were stopped and areas turned into COVID-19 screening area
		Decentralisation of cancer treatment to local and lower-level facilities	Lower-level facilities got overwhelmed with the number of cancer patients
COVID-19 related challenges and unresolved dilemmas	Fear of exposure to COVID-19 infection at the health facility, by both patients and health workers	Lack of PPE	Patient abandonment to cancer services Increased use of simple strategies for infection control such as sanitising working stations and use of hand sanitising gels
		Crowded and congested hospital space and chemotherapy infusion space	
		Lack of room for social distancing/isolation due to open patient wards	
		Lack of readily accessible washing facilities	
		Lack of psychosocial support services to allay fear and anxiety	
	Overall disruption of cancer services spanning the whole cancer control continuum	Travel restrictions	Cancer screening, some surgeries and cancer registration services were halted
		Diversion of already scarce resources (staff and money) to the COVID-19 response	
		Limited access to health facilities	
		Compromised supply chain for cancer drugs, equipment and clinic supplies	
		Lack of electronic data collection systems	
	Challenges of telehealth and technological limitations	Patient discomfort with telehealth and technology	Many patients missed cancer care services during the COVID-19 pandemic
		Slow bandwidth and Internet connectivity	
		High Internet costs and mobile airtime	
		Inadequate skills/capacity in use of telehealth and telecommunication for physical assessments by health workers	
Recommendations for cancer control during and after the COVID-19 pandemic	Health system strengthening	Integrating cancer control services in the health service structures	May strengthen the health system along the entire cancer control continuum that will withstand disruptions during future pandemics.
		Continuing and increasing use of digital technology for cancer control	
		Creating and sharing contextualised guidelines for cancer control in Africa	
		Leveraging the infrastructure and momentum developed in response to COVID-19 pandemic to further strengthen cancer response	
		Developing well-conceived and comprehensive NCCPS	

**Appendix.** Participants of the Africa Cancer ECHO 2019–2020, by geographical distribution.

Country	Number of participants
Austria	4
Botswana	4
WHO AFRO	1
Burundi	2
Cameroon	4
Canada	1
Eswatini	2
Ethiopia	5
Italy	1
Kenya	31
Malawi	8
Namibia	7
Nigeria	5
Rwanda	6
Senegal	1
South Africa	2
Sudan	1
Switzerland	3
Tanzania	4
Uganda	13
USA	47
Zambia	4
Zimbabwe	5
Total	160
